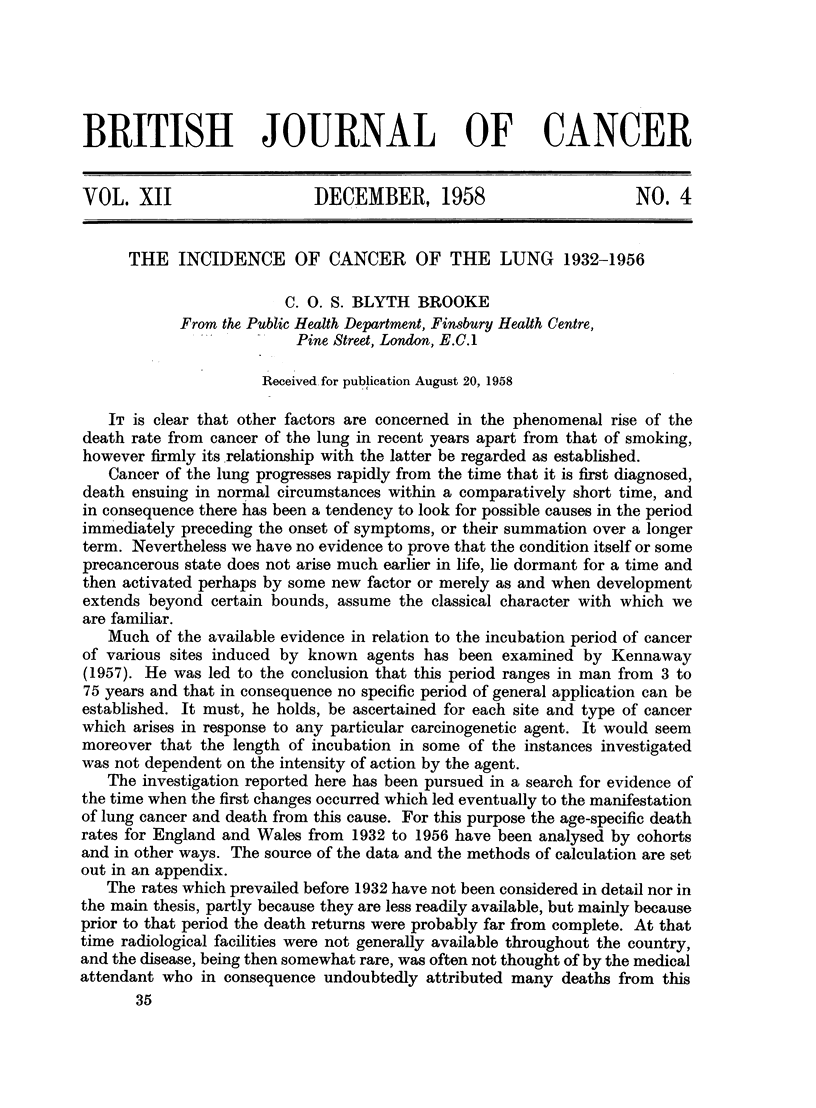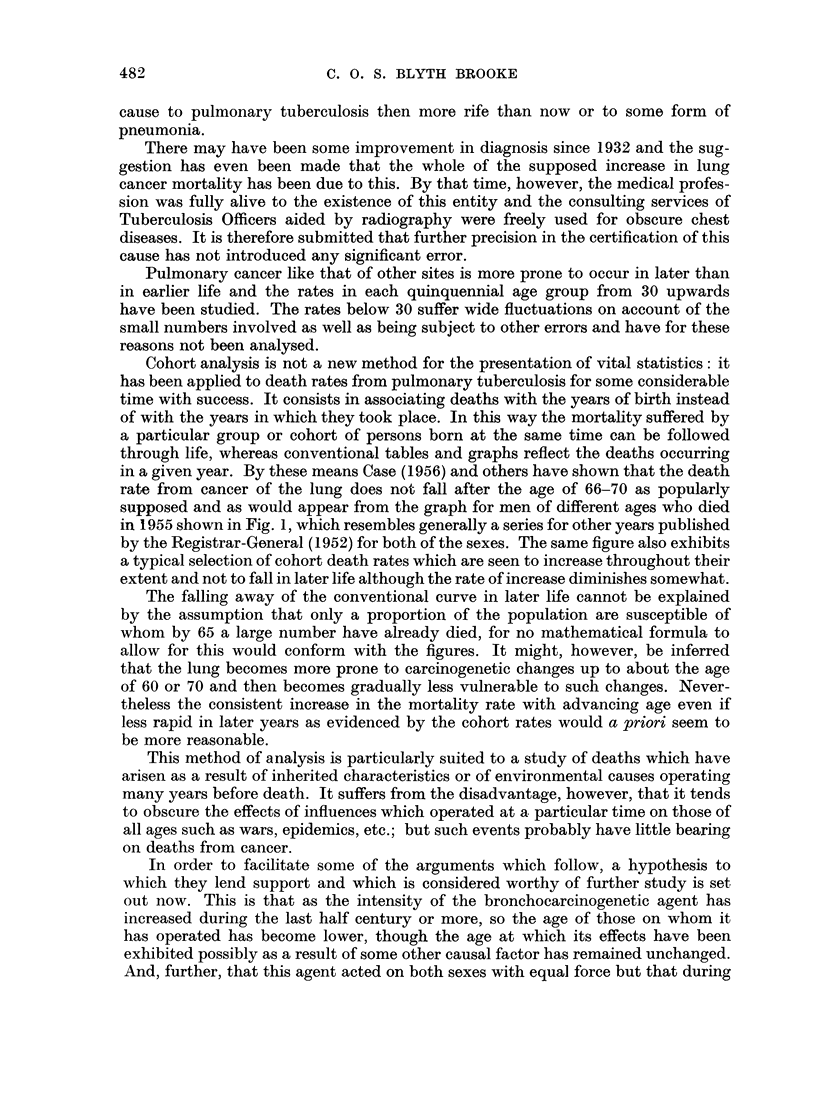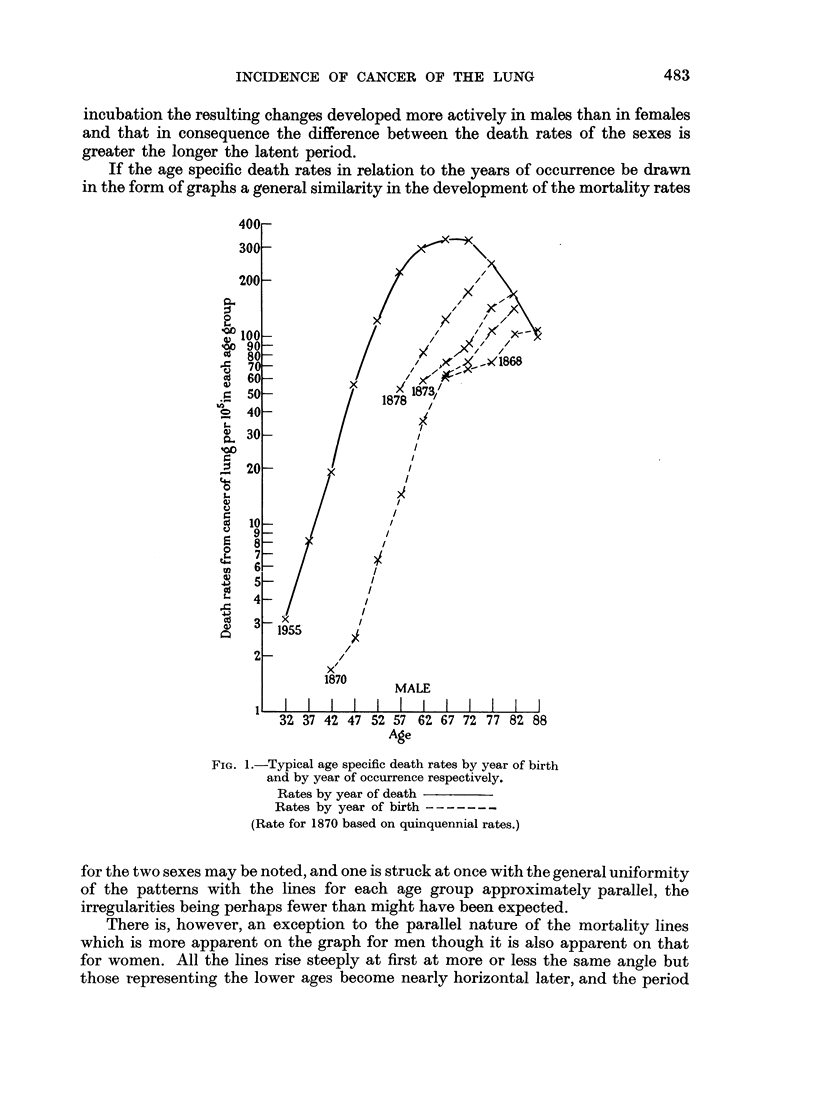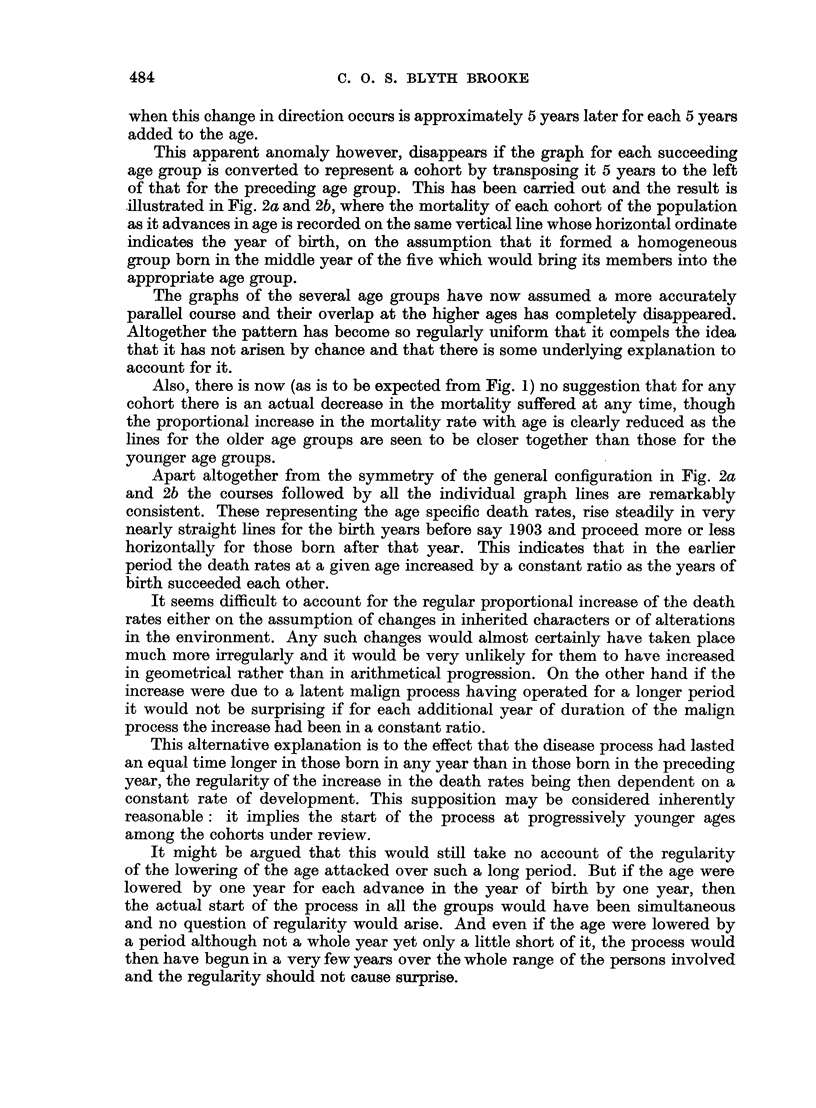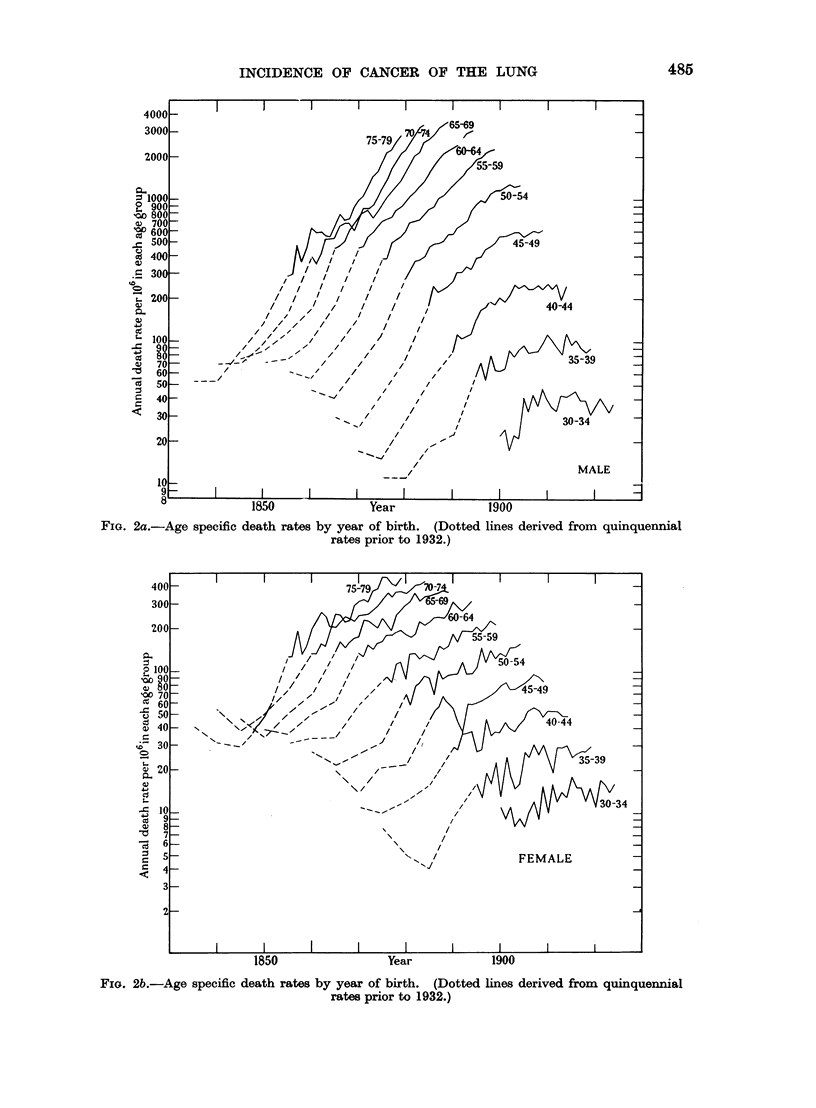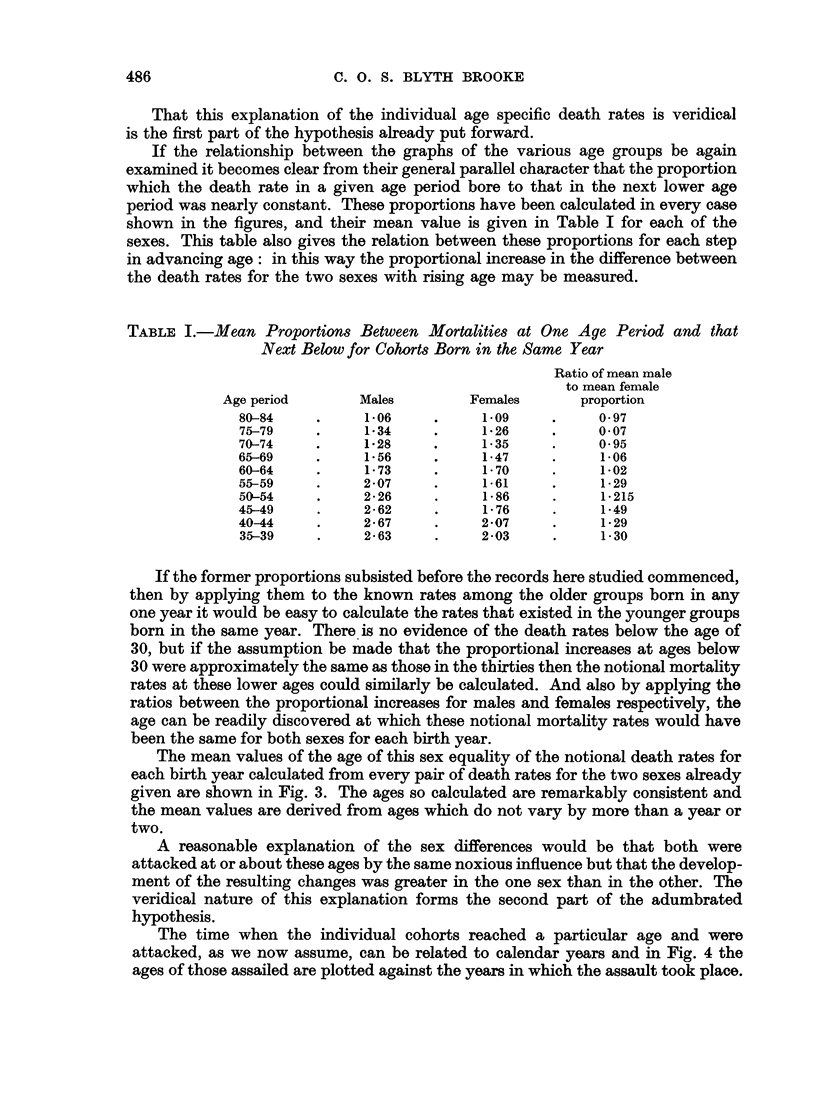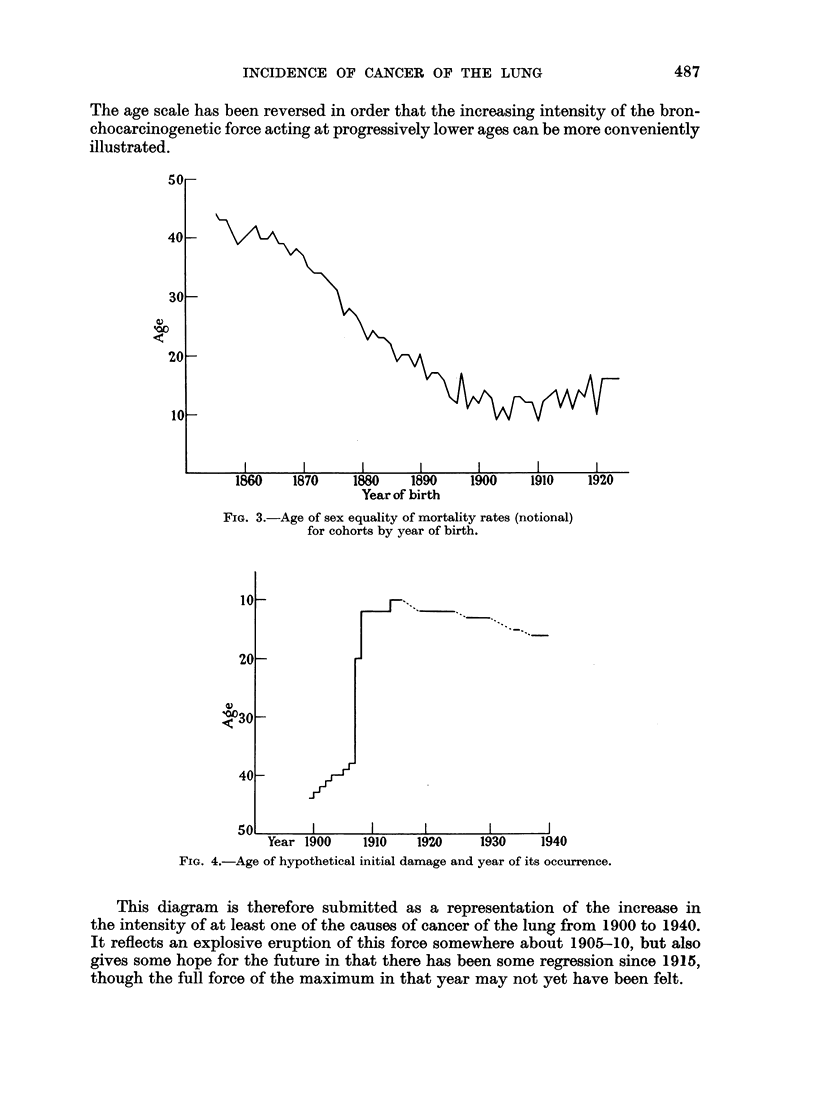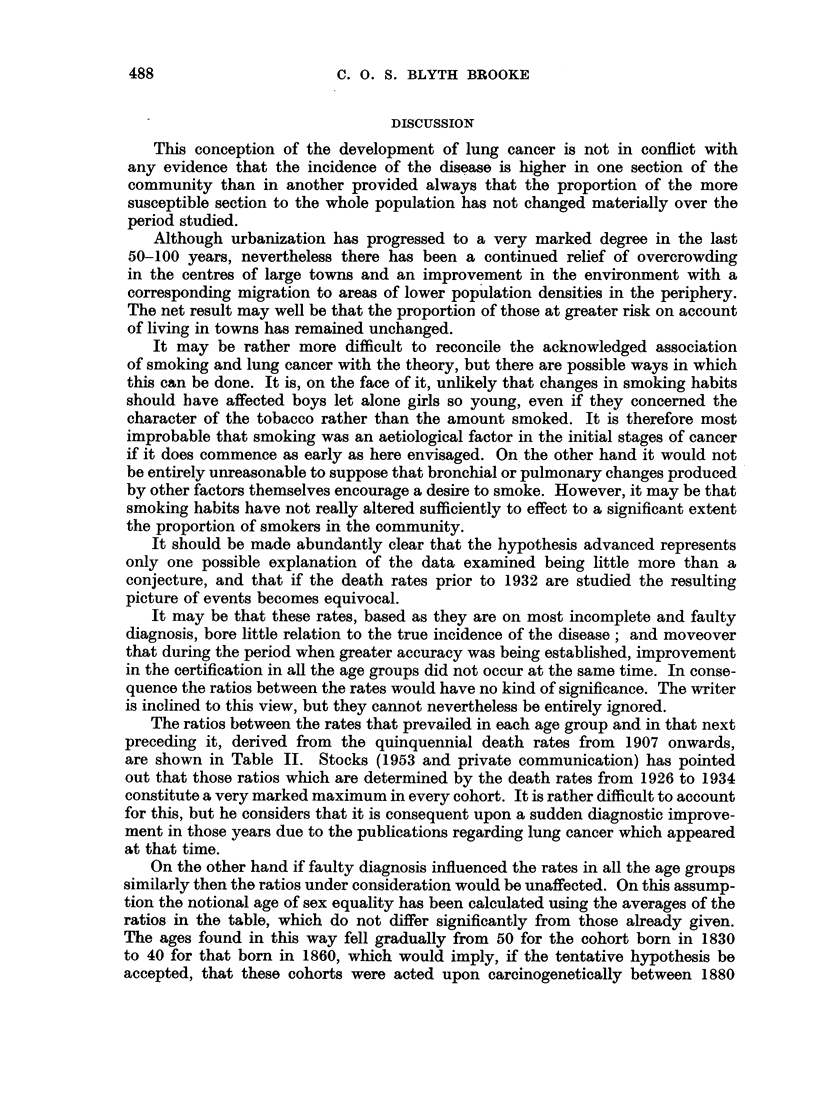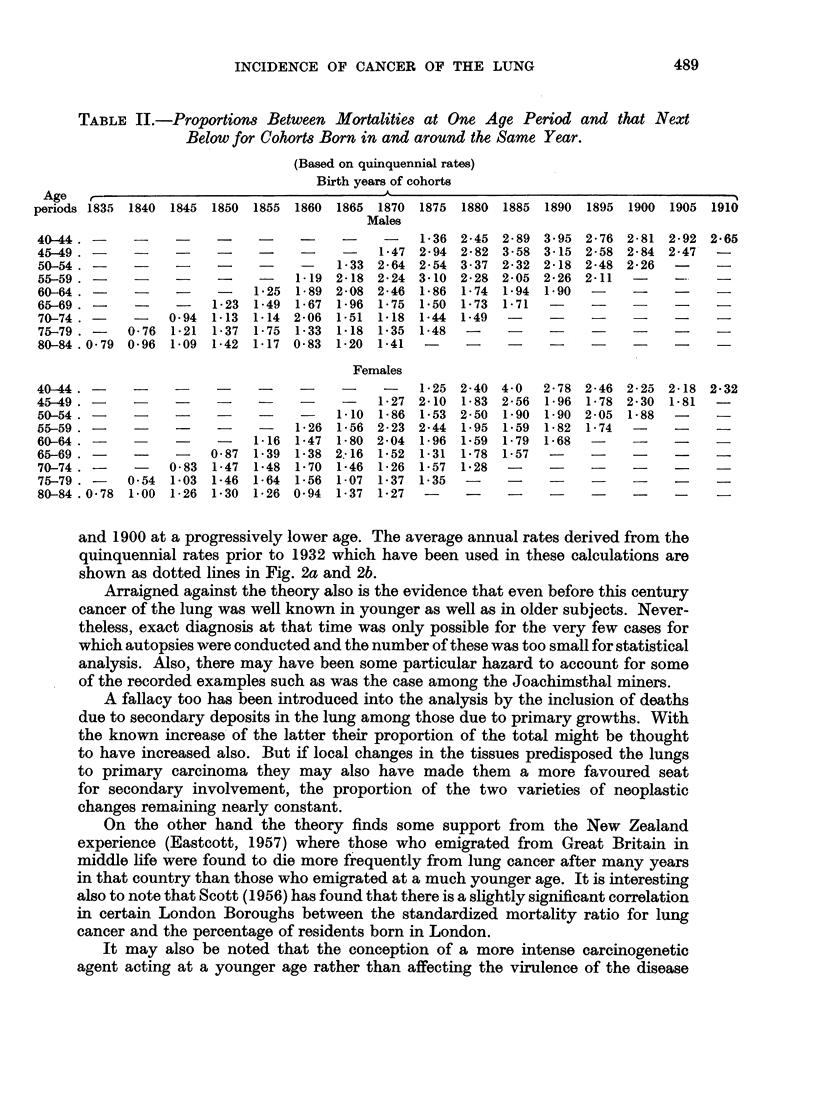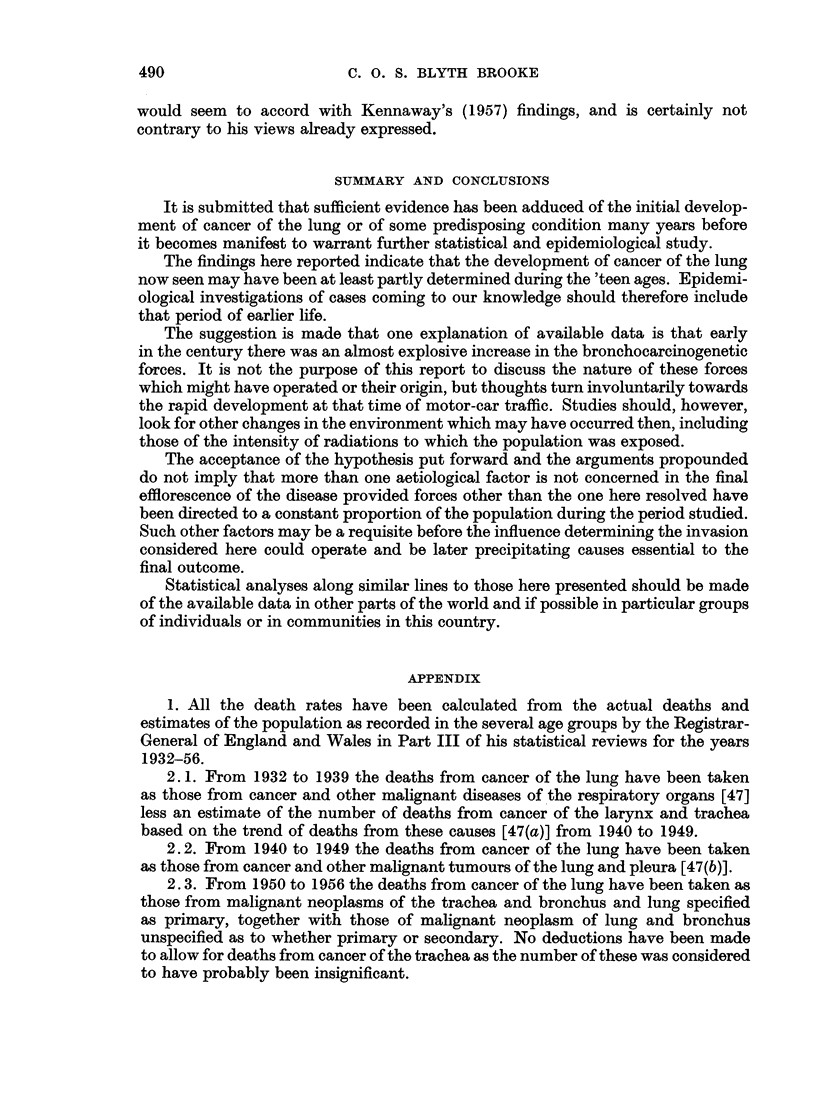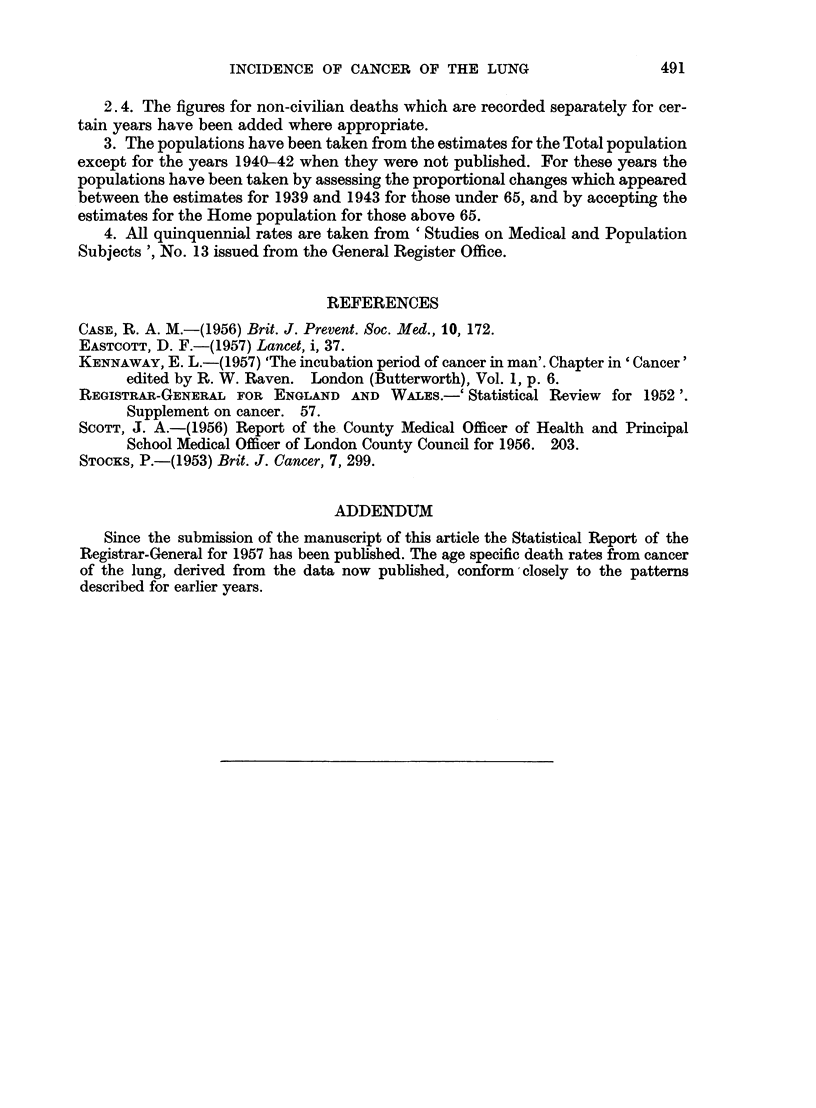# The Incidence of Cancer of the Lung 1932-1956

**DOI:** 10.1038/bjc.1958.56

**Published:** 1958-12

**Authors:** C. O. S. Blyth Brooke


### BRITISH JOURNAL OF CANCER

VOL. XII DECEMBER, 1958 NO. 4

### THE INCIDENCE OF CANCER OF THE LUNG 1932–1956

C. O. S. BLYTH BROOKE

*From the Public Health Department, Finsbury Health Centre, Pine Street, London, E.C.1*

Received for publication August 20, 1958

It is clear that other factors are concerned in the phenomenal rise of the death rate from cancer of the lung in recent years apart from that of smoking, however firmly its relationship with the latter be regarded as established.

Cancer of the lung progresses rapidly from the time that it is first diagnosed, death ensuing in normal circumstances within a comparatively short time, and in consequence there has been a tendency to look for possible causes in the period immediately preceding the onset of symptoms, or their summation over a longer term. Nevertheless we have no evidence to prove that the condition itself or some precancerous state does not arise much earlier in life, lie dormant for a time and then activated perhaps by some new factor or merely as and when development extends beyond certain bounds, assume the classical character with which we are familiar.

Much of the available evidence in relation to the incubation period of cancer of various sites induced by known agents has been examined by Kennaway (1957). He was led to the conclusion that this period ranges in man from 3 to 75 years and that in consequence no specific period of general application can be established. It must, he holds, be ascertained for each site and type of cancer which arises in response to any particular carcinogenetic agent. It would seem moreover that the length of incubation in some of the instances investigated was not dependent on the intensity of action by the agent.

The investigation reported here has been pursued in a search for evidence of the time when the first changes occurred which led eventually to the manifestation of lung cancer and death from this cause. For this purpose the age-specific death rates for England and Wales from 1932 to 1956 have been analysed by cohorts and in other ways. The source of the data and the methods of calculation are set out in an appendix.

The rates which prevailed before 1932 have not been considered in detail nor in the main thesis, partly because they are less readily available, but mainly because prior to that period the death returns were probably far from complete. At that time radiological facilities were not generally available throughout the country, and the disease, being then somewhat rare, was often not thought of by the medical attendant who in consequence undoubtedly attributed many deaths from this cause to pulmonary tuberculosis then more rife than now or to some form of pneumonia.

There may have been some improvement in diagnosis since 1932 and the suggestion has even been made that the whole of the supposed increase in lung cancer mortality has been due to this. By that time, however, the medical profession was fully alive to the existence of this entity and the consulting services of Tuberculosis Officers aided by radiography were freely used for obscure chest diseases. It is therefore submitted that further precision in the certification of this cause has not introduced any significant error.

Pulmonary cancer like that of other sites is more prone to occur in later than in earlier life and the rates in each quinquennial age group from 30 upwards have been studied. The rates below 30 suffer wide fluctuations on account of the small numbers involved as well as being subject to other errors and have for these reasons not been analysed.

Cohort analysis is not a new method for the presentation of vital statistics: it has been applied to death rates from pulmonary tuberculosis for some considerable time with success. It consists in associating deaths with the years of birth instead of with the years in which they took place. In this way the mortality suffered by a particular group or cohort of persons born at the same time can be followed through life, whereas conventional tables and graphs reflect the deaths occurring in a given year. By these means Case (1956) and others have shown that the death rate from cancer of the lung does not fall after the age of 66–70 as popularly supposed and as would appear from the graph for men of different ages who died in 1955 shown in Fig. 1, which resembles generally a series for other years published by the Registrar-General (1952) for both of the sexes. The same figure also exhibits a typical selection of cohort death rates which are seen to increase throughout their extent and not to fall in later life although the rate of increase diminishes somewhat.

The falling away of the conventional curve in later life cannot be explained by the assumption that only a proportion of the population are susceptible of whom by 65 a large number have already died, for no mathematical formula to allow for this would conform with the figures. It might, however, be inferred that the lung becomes more prone to carcinogenetic changes up to about the age of 60 or 70 and then becomes gradually less vulnerable to such changes. Nevertheless the consistent increase in the mortality rate with advancing age even if less rapid in later years as evidenced by the cohort rates would *a priori* seem to be more reasonable.

This method of analysis is particularly suited to a study of deaths which have arisen as a result of inherited characteristics or of environmental causes operating many years before death. It suffers from the disadvantage, however, that it tends to obscure the effects of influences which operated at a particular time on those of all ages such as wars, epidemics, etc.; but such events probably have little bearing on deaths from cancer.

In order to facilitate some of the arguments which follow, a hypothesis to which they lend support and which is considered worthy of further study is set out now. This is that as the intensity of the bronchocarcinogenetic agent has increased during the last half century or more, so the age of those on whom it has operated has become lower, though the age at which its effects have been exhibited possibly as a result of some other causal factor has remained unchanged. And, further, that this agent acted on both sexes with equal force but that during incubation the resulting changes developed more actively in males than in females and that in consequence the difference between the death rates of the sexes is greater the longer the latent period.

If the age specific death rates in relation to the years of occurrence be drawn in the form of graphs a general similarity in the development of the mortality rates

FIG. 1.—Typical age specific death rates by year of birth and by year of occurrence respectively.
Rates by year of death ————
Rates by year of birth – – – – – – –
(Rate for 1870 based on quinquennial rates.)

for the two sexes may be noted, and one is struck at once with the general uniformity of the patterns with the lines for each age group approximately parallel, the irregularities being perhaps fewer than might have been expected.

There is, however, an exception to the parallel nature of the mortality lines which is more apparent on the graph for men though it is also apparent on that for women. All the lines rise steeply at first at more or less the same angle but those representing the lower ages become nearly horizontal later, and the period when this change in direction occurs is approximately 5 years later for each 5 years added to the age.

This apparent anomaly however, disappears if the graph for each succeeding age group is converted to represent a cohort by transposing it 5 years to the left of that for the preceding age group. This has been carried out and the result is illustrated in Fig. 2*a* and 2*b*, where the mortality of each cohort of the population as it advances in age is recorded on the same vertical line whose horizontal ordinate indicates the year of birth, on the assumption that it formed a homogeneous group born in the middle year of the five which would bring its members into the appropriate age group.

The graphs of the several age groups have now assumed a more accurately parallel course and their overlap at the higher ages has completely disappeared. Altogether the pattern has become so regularly uniform that it compels the idea that it has not arisen by chance and that there is some underlying explanation to account for it.

Also, there is now (as is to be expected from Fig. 1) no suggestion that for any cohort there is an actual decrease in the mortality suffered at any time, though the proportional increase in the mortality rate with age is clearly reduced as the lines for the older age groups are seen to be closer together than those for the younger age groups.

Apart altogether from the symmetry of the general configuration in Fig. 2*a* and 2*b* the courses followed by all the individual graph lines are remarkably consistent. These representing the age specific death rates, rise steadily in very nearly straight lines for the birth years before say 1903 and proceed more or less horizontally for those born after that year. This indicates that in the earlier period the death rates at a given age increased by a constant ratio as the years of birth succeeded each other.

It seems difficult to account for the regular proportional increase of the death rates either on the assumption of changes in inherited characters or of alterations in the environment. Any such changes would almost certainly have taken place much more irregularly and it would be very unlikely for them to have increased in geometrical rather than in arithmetical progression. On the other hand if the increase were due to a latent malign process having operated for a longer period it would not be surprising if for each additional year of duration of the malign process the increase had been in a constant ratio.

This alternative explanation is to the effect that the disease process had lasted an equal time longer in those born in any year than in those born in the preceding year, the regularity of the increase in the death rates being then dependent on a constant rate of development. This supposition may be considered inherently reasonable : it implies the start of the process at progressively younger ages among the cohorts under review.

It might be argued that this would still take no account of the regularity of the lowering of the age attacked over such a long period. But if the age were lowered by one year for each advance in the year of birth by one year, then the actual start of the process in all the groups would have been simultaneous and no question of regularity would arise. And even if the age were lowered by a period although not a whole year yet only a little short of it, the process would then have begun in a very few years over the whole range of the persons involved and the regularity should not cause surprise.

FIG. 2*a*.—Age specific death rates by year of birth. (Dotted lines derived from quinquennial rates prior to 1932.)

FIG. 2*b*.—Age specific death rates by year of birth. (Dotted lines derived from quinquennial rates prior to 1932.)

That this explanation of the individual age specific death rates is veridical is the first part of the hypothesis already put forward.

If the relationship between the graphs of the various age groups be again examined it becomes clear from their general parallel character that the proportion which the death rate in a given age period bore to that in the next lower age period was nearly constant. These proportions have been calculated in every case shown in the figures, and their mean value is given in Table I for each of the sexes. This table also gives the relation between these proportions for each step in advancing age : in this way the proportional increase in the difference between the death rates for the two sexes with rising age may be measured.

TABLE I.—*Mean Proportions Between Mortalities at One Age Period and that Next Below for Cohorts Born in the Same Year*

| Age period | Males | Females | Ratio of mean male to mean female proportion |
|---|---|---|---|
| 80–84 | 1·06 | 1·09 | 0·97 |
| 75–79 | 1·34 | 1·26 | 0·07 |
| 70–74 | 1·28 | 1·35 | 0·95 |
| 65–69 | 1·56 | 1·47 | 1·06 |
| 60–64 | 1·73 | 1·70 | 1·02 |
| 55–59 | 2·07 | 1·61 | 1·29 |
| 50–54 | 2·26 | 1·86 | 1·215 |
| 45–49 | 2·62 | 1·76 | 1·49 |
| 40–44 | 2·67 | 2·07 | 1·29 |
| 35–39 | 2·63 | 2·03 | 1·30 |

If the former proportions subsisted before the records here studied commenced, then by applying them to the known rates among the older groups born in any one year it would be easy to calculate the rates that existed in the younger groups born in the same year. There is no evidence of the death rates below the age of 30, but if the assumption be made that the proportional increases at ages below 30 were approximately the same as those in the thirties then the notional mortality rates at these lower ages could similarly be calculated. And also by applying the ratios between the proportional increases for males and females respectively, the age can be readily discovered at which these notional mortality rates would have been the same for both sexes for each birth year.

The mean values of the age of this sex equality of the notional death rates for each birth year calculated from every pair of death rates for the two sexes already given are shown in Fig. 3. The ages so calculated are remarkably consistent and the mean values are derived from ages which do not vary by more than a year or two.

A reasonable explanation of the sex differences would be that both were attacked at or about these ages by the same noxious influence but that the development of the resulting changes was greater in the one sex than in the other. The veridical nature of this explanation forms the second part of the adumbrated hypothesis.

The time when the individual cohorts reached a particular age and were attacked, as we now assume, can be related to calendar years and in Fig. 4 the ages of those assailed are plotted against the years in which the assault took place.

The age scale has been reversed in order that the increasing intensity of the bronchocarcinogenetic force acting at progressively lower ages can be more conveniently illustrated.

FIG. 3.—Age of sex equality of mortality rates (notional) for cohorts by year of birth.

FIG. 4.—Age of hypothetical initial damage and year of its occurrence.

This diagram is therefore submitted as a representation of the increase in the intensity of at least one of the causes of cancer of the lung from 1900 to 1940. It reflects an explosive eruption of this force somewhere about 1905–10, but also gives some hope for the future in that there has been some regression since 1915, though the full force of the maximum in that year may not yet have been felt.

### DISCUSSION

This conception of the development of lung cancer is not in conflict with any evidence that the incidence of the disease is higher in one section of the community than in another provided always that the proportion of the more susceptible section to the whole population has not changed materially over the period studied.

Although urbanization has progressed to a very marked degree in the last 50–100 years, nevertheless there has been a continued relief of overcrowding in the centres of large towns and an improvement in the environment with a corresponding migration to areas of lower population densities in the periphery. The net result may well be that the proportion of those at greater risk on account of living in towns has remained unchanged.

It may be rather more difficult to reconcile the acknowledged association of smoking and lung cancer with the theory, but there are possible ways in which this can be done. It is, on the face of it, unlikely that changes in smoking habits should have affected boys let alone girls so young, even if they concerned the character of the tobacco rather than the amount smoked. It is therefore most improbable that smoking was an aetiological factor in the initial stages of cancer if it does commence as early as here envisaged. On the other hand it would not be entirely unreasonable to suppose that bronchial or pulmonary changes produced by other factors themselves encourage a desire to smoke. However, it may be that smoking habits have not really altered sufficiently to effect to a significant extent the proportion of smokers in the community.

It should be made abundantly clear that the hypothesis advanced represents only one possible explanation of the data examined being little more than a conjecture, and that if the death rates prior to 1932 are studied the resulting picture of events becomes equivocal.

It may be that these rates, based as they are on most incomplete and faulty diagnosis, bore little relation to the true incidence of the disease ; and moveover that during the period when greater accuracy was being established, improvement in the certification in all the age groups did not occur at the same time. In consequence the ratios between the rates would have no kind of significance. The writer is inclined to this view, but they cannot nevertheless be entirely ignored.

The ratios between the rates that prevailed in each age group and in that next preceding it, derived from the quinquennial death rates from 1907 onwards, are shown in Table II. Stocks (1953 and private communication) has pointed out that those ratios which are determined by the death rates from 1926 to 1934 constitute a very marked maximum in every cohort. It is rather difficult to account for this, but he considers that it is consequent upon a sudden diagnostic improvement in those years due to the publications regarding lung cancer which appeared at that time.

On the other hand if faulty diagnosis influenced the rates in all the age groups similarly then the ratios under consideration would be unaffected. On this assumption the notional age of sex equality has been calculated using the averages of the ratios in the table, which do not differ significantly from those already given. The ages found in this way fell gradually from 50 for the cohort born in 1830 to 40 for that born in 1860, which would imply, if the tentative hypothesis be accepted, that these cohorts were acted upon carcinogenetically between 1880

TABLE II.—*Proportions Between Mortalities at One Age Period and that Next Below for Cohorts Born in and around the Same Year.*

(Based on quinquennial rates)

| Age periods | Birth years of cohorts | | | | | | | | | | | | | | | |
|---|---|---|---|---|---|---|---|---|---|---|---|---|---|---|---|---|
| | 1835 | 1840 | 1845 | 1850 | 1855 | 1860 | 1865 | 1870 | 1875 | 1880 | 1885 | 1890 | 1895 | 1900 | 1905 | 1910 |
| | | | | | | | | Males | | | | | | | | |
| 40–44 . | — | — | — | — | — | — | — | — | 1·36 | 2·45 | 2·89 | 3·95 | 2·76 | 2·81 | 2·92 | 2·65 |
| 45–49 . | — | — | — | — | — | — | — | 1·47 | 2·94 | 2·82 | 3·58 | 3·15 | 2·58 | 2·84 | 2·47 | — |
| 50–54 . | — | — | — | — | — | — | 1·33 | 2·64 | 2·54 | 3·37 | 2·32 | 2·18 | 2·48 | 2·26 | — | — |
| 55–59 . | — | — | — | — | — | 1·19 | 2·18 | 2·24 | 3·10 | 2·28 | 2·05 | 2·26 | 2·11 | — | — | — |
| 60–64 . | — | — | — | — | 1·25 | 1·89 | 2·08 | 2·46 | 1·86 | 1·74 | 1·94 | 1·90 | — | — | — | — |
| 65–69 . | — | — | — | 1·23 | 1·49 | 1·67 | 1·96 | 1·75 | 1·50 | 1·73 | 1·71 | — | — | — | — | — |
| 70–74 . | — | — | 0·94 | 1·13 | 1·14 | 2·06 | 1·51 | 1·18 | 1·44 | 1·49 | — | — | — | — | — | — |
| 75–79 . | — | 0·76 | 1·21 | 1·37 | 1·75 | 1·33 | 1·18 | 1·35 | 1·48 | — | — | — | — | — | — | — |
| 80–84 . | 0·79 | 0·96 | 1·09 | 1·42 | 1·17 | 0·83 | 1·20 | 1·41 | — | — | — | — | — | — | — | — |
| | | | | | | | | Females | | | | | | | | |
| 40–44 . | — | — | — | — | — | — | — | — | 1·25 | 2·40 | 4·0 | 2·78 | 2·46 | 2·25 | 2·18 | 2·32 |
| 45–49 . | — | — | — | — | — | — | — | 1·27 | 2·10 | 1·83 | 2·56 | 1·96 | 1·78 | 2·30 | 1·81 | — |
| 50–54 . | — | — | — | — | — | — | 1·10 | 1·86 | 1·53 | 2·50 | 1·90 | 1·90 | 2·05 | 1·88 | — | — |
| 55–59 . | — | — | — | — | — | 1·26 | 1·56 | 2·23 | 2·44 | 1·95 | 1·59 | 1·82 | 1·74 | — | — | — |
| 60–64 . | — | — | — | — | 1·16 | 1·47 | 1·80 | 2·04 | 1·96 | 1·59 | 1·79 | 1·68 | — | — | — | — |
| 65–69 . | — | — | — | 0·87 | 1·39 | 1·38 | 2·16 | 1·52 | 1·31 | 1·78 | 1·57 | — | — | — | — | — |
| 70–74 . | — | — | 0·83 | 1·47 | 1·48 | 1·70 | 1·46 | 1·26 | 1·57 | 1·28 | — | — | — | — | — | — |
| 75–79 . | — | 0·54 | 1·03 | 1·46 | 1·64 | 1·56 | 1·07 | 1·37 | 1·35 | — | — | — | — | — | — | — |
| 80–84 . | 0·78 | 1·00 | 1·26 | 1·30 | 1·26 | 0·94 | 1·37 | 1·27 | — | — | — | — | — | — | — | — |

and 1900 at a progressively lower age. The average annual rates derived from the quinquennial rates prior to 1932 which have been used in these calculations are shown as dotted lines in Fig. 2*a* and 2*b*.

Arraigned against the theory also is the evidence that even before this century cancer of the lung was well known in younger as well as in older subjects. Nevertheless, exact diagnosis at that time was only possible for the very few cases for which autopsies were conducted and the number of these was too small for statistical analysis. Also, there may have been some particular hazard to account for some of the recorded examples such as was the case among the Joachimsthal miners.

A fallacy too has been introduced into the analysis by the inclusion of deaths due to secondary deposits in the lung among those due to primary growths. With the known increase of the latter their proportion of the total might be thought to have increased also. But if local changes in the tissues predisposed the lungs to primary carcinoma they may also have made them a more favoured seat for secondary involvement, the proportion of the two varieties of neoplastic changes remaining nearly constant.

On the other hand the theory finds some support from the New Zealand experience (Eastcott, 1957) where those who emigrated from Great Britain in middle life were found to die more frequently from lung cancer after many years in that country than those who emigrated at a much younger age. It is interesting also to note that Scott (1956) has found that there is a slightly significant correlation in certain London Boroughs between the standardized mortality ratio for lung cancer and the percentage of residents born in London.

It may also be noted that the conception of a more intense carcinogenetic agent acting at a younger age rather than affecting the virulence of the disease would seem to accord with Kennaway's (1957) findings, and is certainly not contrary to his views already expressed.

### SUMMARY AND CONCLUSIONS

It is submitted that sufficient evidence has been adduced of the initial development of cancer of the lung or of some predisposing condition many years before it becomes manifest to warrant further statistical and epidemiological study.

The findings here reported indicate that the development of cancer of the lung now seen may have been at least partly determined during the 'teen ages. Epidemiological investigations of cases coming to our knowledge should therefore include that period of earlier life.

The suggestion is made that one explanation of available data is that early in the century there was an almost explosive increase in the bronchocarcinogenetic forces. It is not the purpose of this report to discuss the nature of these forces which might have operated or their origin, but thoughts turn involuntarily towards the rapid development at that time of motor-car traffic. Studies should, however, look for other changes in the environment which may have occurred then, including those of the intensity of radiations to which the population was exposed.

The acceptance of the hypothesis put forward and the arguments propounded do not imply that more than one aetiological factor is not concerned in the final efflorescence of the disease provided forces other than the one here resolved have been directed to a constant proportion of the population during the period studied. Such other factors may be a requisite before the influence determining the invasion considered here could operate and be later precipitating causes essential to the final outcome.

Statistical analyses along similar lines to those here presented should be made of the available data in other parts of the world and if possible in particular groups of individuals or in communities in this country.

### APPENDIX

1. All the death rates have been calculated from the actual deaths and estimates of the population as recorded in the several age groups by the Registrar-General of England and Wales in Part III of his statistical reviews for the years 1932–56.

2.1. From 1932 to 1939 the deaths from cancer of the lung have been taken as those from cancer and other malignant diseases of the respiratory organs [47] less an estimate of the number of deaths from cancer of the larynx and trachea based on the trend of deaths from these causes [47(*a*)] from 1940 to 1949.

2.2. From 1940 to 1949 the deaths from cancer of the lung have been taken as those from cancer and other malignant tumours of the lung and pleura [47(*b*)].

2.3. From 1950 to 1956 the deaths from cancer of the lung have been taken as those from malignant neoplasms of the trachea and bronchus and lung specified as primary, together with those of malignant neoplasm of lung and bronchus unspecified as to whether primary or secondary. No deductions have been made to allow for deaths from cancer of the trachea as the number of these was considered to have probably been insignificant.

2.4. The figures for non-civilian deaths which are recorded separately for certain years have been added where appropriate.

3. The populations have been taken from the estimates for the Total population except for the years 1940–42 when they were not published. For these years the populations have been taken by assessing the proportional changes which appeared between the estimates for 1939 and 1943 for those under 65, and by accepting the estimates for the Home population for those above 65.

4. All quinquennial rates are taken from ' Studies on Medical and Population Subjects ', No. 13 issued from the General Register Office.

### REFERENCES

CASE, R. A. M.—(1956) *Brit. J. Prevent. Soc. Med.*, **10**, 172.

EASTCOTT, D. F.—(1957) *Lancet*, i, 37.

KENNAWAY, E. L.—(1957) 'The incubation period of cancer in man'. Chapter in ' Cancer ' edited by R. W. Raven. London (Butterworth), Vol. 1, p. 6.

REGISTRAR-GENERAL FOR ENGLAND AND WALES.—' Statistical Review for 1952 '. Supplement on cancer. 57.

SCOTT, J. A.—(1956) Report of the County Medical Officer of Health and Principal School Medical Officer of London County Council for 1956. 203.

STOCKS, P.—(1953) *Brit. J. Cancer*, **7**, 299.

### ADDENDUM

Since the submission of the manuscript of this article the Statistical Report of the Registrar-General for 1957 has been published. The age specific death rates from cancer of the lung, derived from the data now published, conform closely to the patterns described for earlier years.